# Artificial intelligence readiness and its association with artificial intelligence literacy among Chinese medical students: a latent profile analysis

**DOI:** 10.3389/fpubh.2026.1963947

**Published:** 2026-09-23

**Authors:** Zongsheng Tang, Shuhong Song, Xinyue Chen, Jiaxu Qin, Yan Li, Jingjing Yu, Chen Yu, Huan Liu, Xiangxiang Tao

**Affiliations:** 1 Department of Transfusion Medicine, The First Affiliated Hospital of Wannan Medical University (Yijishan Hospital of Wannan Medical University), Wuhu, Anhui, China; 2 Graduate School, Wannan Medical University, Wuhu, Anhui, China; 3 Department of Hemodialysis, The First Affiliated Hospital of Wannan Medical University (Yijishan Hospital of Wannan Medical University), Wuhu, Anhui, China; 4 School of Clinical Medicine, Wannan Medical University, Wuhu, Anhui, China

**Keywords:** artificial intelligence literacy, artificial intelligence readiness, latent profile analysis, medical education, medical students

## Abstract

**Objectives:**

To identify heterogeneous subgroups of medical students’ artificial intelligence readiness through latent profile analysis and to examine their associations with artificial intelligence literacy.

**Methods:**

A cross-sectional survey was conducted from February to May 2026 among 707 medical students from four universities in Anhui Province, China, using the Medical Artificial Intelligence Readiness Scale and the Artificial Intelligence Literacy Scale. Latent profile analysis was performed using the 22 artificial intelligence readiness items as manifest indicators. Model selection was based on information criteria, entropy, likelihood-ratio tests, profile size, posterior classification probabilities, parsimony, and interpretability. Chi-square tests, one-way analysis of variance, and multinomial logistic regression were used for exploratory profile comparisons.

**Results:**

Latent profile analysis identified three distinct artificial intelligence readiness profiles: low (*n* = 342, 48.4%), moderate (*n* = 293, 41.4%), and high (*n* = 72, 10.2%). Multinomial logistic regression revealed that frequent exposure to artificial intelligence-medicine interdisciplinary courses, interest in artificial intelligence, and higher satisfaction with current major were significant predictors of belonging to higher readiness profiles. A significant graded association emerged, with high-readiness students demonstrating substantially superior artificial intelligence literacy across all dimensions compared to their moderate- and low-readiness counterparts.

**Conclusion:**

Medical students exhibit substantial heterogeneity in artificial intelligence readiness, with nearly half demonstrating low preparedness. The pronounced deficits in practical ability and the strong linkage between readiness profiles and comprehensive artificial intelligence literacy underscore the urgent need for tiered, behavior-oriented curricular interventions that foster proactive engagement and hands-on artificial intelligence skills across the continuum of medical education.

## 1 Introduction

Artificial intelligence (AI) is rapidly transforming the global healthcare landscape and medical education, with its integration into clinical practice offering the potential to enhance diagnostic accuracy, optimize treatment planning, and streamline administrative workflows. Concurrently, AI-driven educational tools, including simulation platforms and wearable sensor systems, are reshaping pedagogical approaches for training future healthcare professionals, providing novel avenues for skill acquisition and objective performance assessment (1, 2). In this evolving context, the role of physicians is being redefined, necessitating competencies that extend beyond traditional clinical knowledge. Medical educators and policymakers thus face the critical challenge of preparing a workforce capable of effectively and ethically navigating AI-enhanced clinical environments. Given this paradigm shift, there is a pressing need to systematically evaluate and cultivate AI readiness among medical students, ensuring graduates are equipped to meet the demands of a technologically advanced healthcare system.

Globally, the imperative to equip medical students with AI competencies is now widely recognized, with numerous countries integrating AI literacy into core competency frameworks for medical education (3, 4). This shift reflects the profound and accelerating influence of AI on clinical practice, where tools such as machine learning and generative models are reshaping diagnostic processes and patient care workflows. However, despite this top-down acknowledgment, a significant disconnect persists at the implementation level. Traditional medical curricula, historically grounded in a balanced emphasis on medical humanities and clinical skills, have not yet systematically responded to the need to cultivate this novel competency domain (5). While various frameworks and consensus statements define what AI literacy should entail, empirical evidence indicates that its integration into formal educational structures remains limited and fragmented. This gap between the recognized necessity of AI readiness and the actual preparedness of the student body necessitates a granular exploration of how medical students are currently positioned to engage with AI, particularly within systems where curricular reform is still nascent.

In this context, AI readiness has emerged as a critical area of inquiry, encompassing not only cognitive understanding and technical ability but also practical skills, visionary outlook, and ethical considerations necessary for responsible application in patient care (6, 7). A foundational grasp of AI and a positive attitude toward its role as a supportive tool are increasingly recognized as essential components of modern medical training (8). While students frequently express enthusiasm for incorporating AI into their future practice, many also report a perceived lack of structured education and formal training, which may contribute to anxieties about its professional impact (9). Studies across different countries have consistently revealed that formal training remains insufficient, with a substantial proportion of students expressing concerns about ethical issues, reduced independent thinking, and limited practical exposure (10, 11). This rapid integration of AI into clinical practice and medical education has thus created an urgent need for medical students to be adequately prepared for its application (12). It should be noted that, although closely related, AI readiness and AI literacy are conceptually distinct constructs. AI literacy refers to the set of competencies that enable individuals to identify AI technologies, to use AI applications or products, to evaluate their capabilities and limitations, and to apply them in an ethically compliant manner, whereas AI readiness denotes a broader multidimensional state of preparedness for the application of AI in healthcare, encompassing not only such competencies but also cognitive understanding, practical ability, visionary outlook, and ethical awareness (3, 6, 7). Accordingly, the present study treated AI readiness as the focal classification construct and AI literacy as a related competency outcome.

While aggregate scores provide a general indication of preparedness, they may obscure meaningful structural differences across distinct subgroups of students. Recent evidence suggests that students exhibit heterogeneous patterns across these dimensions, with some demonstrating strengths in ethical considerations while lagging in practical skills or cognitive foundations (13, 14). Such variability implies that a singular mean score cannot adequately capture the nuanced readiness profiles likely to exist within a diverse student body. Consequently, relying solely on overall measurements could mask the specific educational needs of different learner groups. To address this complexity and move beyond variable-centered approaches, a person-centered analytical method, such as latent profile analysis (LPA) (15, 16), is warranted. LPA enables the identification of unobserved, homogeneous subgroups characterized by distinct combinations of AI readiness dimensions, thereby facilitating the design of tailored educational interventions that more precisely match the diverse preparation levels found among medical students (17, 18).

This person-centered approach offers a distinct methodological advantage by revealing qualitative differences in AI readiness that are not captured by variable-centered methods, and it has been effectively applied in other areas of medical education to differentiate learner subtypes and tailor pedagogical strategies. Within the specific domain of medical students’ AI readiness, despite a growing body of research exploring its prevalence and associated factors (13, 19). The application of LPA to identify such subpopulations remains conspicuously underexplored. To the best of our knowledge and based on our literature search, however, few published studies have yet employed this approach to characterize distinct AI readiness profiles among medical students, and no such study has been identified in the context of Chinese medical education.

While readiness profiles delineate distinct levels of preparedness, their alignment with actual competencies, such as AI literacy, remains insufficiently explored, particularly within medical education (20). Existing research has established a correlation between AI literacy and positive attitudes toward digital technology among health profession students, suggesting that higher literacy may mitigate technology-related stress (21). Furthermore, studies indicate that AI literacy can strengthen the relationship between AI usage and learning outcomes, highlighting its importance in educational settings (22). However, these investigations often treat learners as a homogeneous group, overlooking the possibility of distinct readiness subpopulations that may exhibit varying literacy levels. Given the recognized need for curricula to cultivate critical appraisal skills and adapt to evolving AI technologies (23, 24) understanding how different readiness strata correspond to literacy is essential. Therefore, using an LPA approach with medical students from multiple universities in Anhui Province, this study aimed to identify potential classes of medical students’ AI readiness, clarify the demographic characteristics and distribution proportions of each subgroup, and further examine differences in AI literacy across identified readiness classes. This will reveal the gradient association between readiness stratification and literacy levels, thereby offering empirical evidence for refining medical AI curriculum design and implementing stratified instructional strategies.

## 2 Materials and methods

### 2.1 Study design and participants

This study employed a cross-sectional design. Using convenience sampling, medical students were recruited from one provincial key university and three provincial regular universities in Anhui Province between February 2026 and May 2026. These universities differed in location, teaching strength, and student population, resulting in variations in medical students’ access to learning resources. Inclusion criteria were: (1) full-time undergraduate medical students currently enrolled; (2) no history of mental illness or use of psychiatric medications; (3) voluntary participation in this study. Exclusion criteria were: (1) medical students who were on leave or had withdrawn from school during the survey period; (2) incomplete questionnaire responses. Of note, the mental-health-related criteria were operationalized through self-report on the general information questionnaire: participants indicated whether they had a physician-diagnosed history of mental illness or were currently taking psychiatric medications, and those responding affirmatively were screened out (*n* = 4). The criterion was applied because both instruments rely on self-reported psychological and perceptual evaluations, the reliability of which may be compromised among students with acute psychiatric conditions or those taking psychotropic medication; the potential selection bias introduced by this criterion is acknowledged in the limitations.

LPA requires a minimum sample size of 300 participants to ensure the robustness and precision of subgroup classification results (15). This threshold is consistent with simulation evidence showing that samples of approximately 300 or more provide adequate precision for identifying medium-sized latent classes, whereas samples exceeding 500 further improve the stability of smaller classes (16). Considering the possibility of missing data or invalid questionnaires and assuming an invalidity rate of 10%, the estimated sample size required for this study was at least 330 cases. The final sample of 707 participants exceeded this requirement and provided adequate precision and stability for the three-profile solution, including the smallest profile (*n* = 72), whose size remained sufficient for stable class-specific estimation (15).

### 2.2 Measures

#### 2.2.1 General information questionnaire

Based on literature review and research objectives, this was self-designed by the team, including 13 items: gender, place of origin, class cadre, only child, grade level, self-rated previous academic performance, voluntarily chosen the major, exposure to AI-medicine interdisciplinary courses, received AI training, interest in AI, frequency of AI tool use, satisfaction with current major, and major.

#### 2.2.2 Chinese version of the medical artificial intelligence readiness scale for medical students (C-MAIRS-MS)

This scale was developed by Karaca et al. (25) in 2021 and translated into Chinese by Xuancheng Chen et al. (26). in 2025. It is used to assess medical students’ artificial intelligence readiness, including four dimensions: cognition (6 items), ability (10 items), vision (3 items), and ethics (3 items), comprising 22 items in total. A Likert 5-point scoring method was adopted, with “strongly disagree, disagree, neutral, agree, strongly agree” scored from 1 to 5 points, respectively. The total score ranges from 22 to 110, with higher scores indicating higher medical artificial intelligence readiness. The Cronbach’s *α* coefficient of this scale was 0.968. The specific content of the 22 items is presented in Supplementary Table S1.

#### 2.2.3 Artificial intelligence literacy scale (AIL)

Developed by Wang et al. (27) in 2023, this scale includes four dimensions: awareness, use, evaluation, and ethics. Each dimension has 3 items, totaling 12 items. The scale adopts a Likert 7-point scoring method (1 = strongly disagree, 7 = strongly agree). The total score ranges from 12 to 84, with higher scores indicating higher AI literacy levels. The Cronbach’s *α* coefficients for the dimensions ranged from 0.73 to 0.78, and the overall Cronbach’s *α* coefficient of this scale was 0.860. The specific content of the 12 items is presented in Supplementary Table S2.

### 2.3 Data collection

Data were collected online through the Wenjuanxing platform (a widely used online survey platform in China). Each questionnaire was accompanied by an informed consent form, and questionnaires were only completed after obtaining the participants’ consent. The survey took approximately 10–15 min. Each device and IP address was restricted to one response only, and all items were required to be completed before submission. Questionnaires with a completion time of less than 300 s or showing obvious response patterns were excluded.

### 2.4 Statistical analysis

Mplus 8.3 was used to conduct LPA of medical students’ artificial intelligence readiness, with the 22 items of the MAIRS-MS as observed indicators. The number of latent classes was increased sequentially from a one-class model. Model fit indices were evaluated: Akaike Information Criterion (AIC), Bayesian Information Criterion (BIC), and adjusted BIC (aBIC), with smaller values indicating better fit (16); Entropy values closer to 1 indicated more accurate classification. Likelihood ratio tests, including the Lo–Mendell–Rubin (LMR) test and the Bootstrap Likelihood Ratio Test (BLRT) (28), were used to compare models, with *p* < 0.05 indicating that the k-class model was superior to the k-1 class model (29). In specifying the models, the 22 item scores were treated as continuous indicators following normal distributions within each latent profile; residual variances were constrained to be equal across profiles, and covariances among indicators were fixed to zero (i.e., local independence was assumed). Models were estimated using maximum likelihood with 500 random sets of starting values and 100 final-stage optimizations to guard against local maxima, and the best log-likelihood value was successfully replicated for each model. Because all questionnaire items were mandatory, no missing data were present on the model indicators.

SPSS 26.0 was used for statistical analysis. Continuous variables were described as mean ± standard deviation or median with interquartile range; categorical variables were described as frequencies and constituent ratios. Differences in distribution across different classes of medical students’ artificial intelligence readiness were tested using the chi-square test. Factors influencing latent classes of artificial intelligence readiness were analyzed using multinomial logistic regression. Differences in artificial intelligence literacy across latent classes were compared using one-way analysis of variance (ANOVA) with *post hoc* pairwise tests. The three-profile solution showed relatively high classification quality in the present sample (entropy = 0.986; average posterior probabilities ≥ 0.986). It should be noted, however, that entropy and posterior probabilities are sample-specific descriptive indices; although such values markedly reduce concerns about classification error, they cannot entirely preclude misclassification of individual participants. Accordingly, a classify-analyze strategy was adopted in which participants were assigned to their most likely profile, and the potential underestimation of standard errors arising from classification uncertainty is acknowledged in the limitations. The significance level was set at *α* = 0.05.

## 3 Results

### 3.1 General characteristics of medical students participating in the survey

A total of 707 medical students were included, including 310 males and 397 females. Place of origin: rural (*n* = 390), town (*n* = 197), urban (*n* = 120). Class cadre: yes (*n* = 197), no (*n* = 510). Only child: yes (*n* = 180), no (*n* = 527). Grade level: first year (*n* = 212), second year (*n* = 164), third year (*n* = 186), fourth year (*n* = 74), fifth year (*n* = 71). Self-rated academic performance: poor (*n* = 118), average (*n* = 211), good (*n* = 299), excellent (*n* = 79). Exposure to AI-medicine interdisciplinary courses: never (*n* = 43), rarely (*n* = 128), occasionally (*n* = 381), frequently (*n* = 107), very frequently (*n* = 48). Interest in AI: yes (*n* = 523), no (*n* = 184). More detailed participant information is presented in Table 1.

**Table 1:** Participant characteristics (*n* = 707).

Variable	Category	*n*	Percentage (%)
Gender			
	Male	310	43.84
	Female	397	56.16
Place of origin			
	Rural	390	55.16
	Town	197	27.86
	Urban	120	16.97
Class cadre			
	Yes	197	27.86
	No	510	72.14
Only child			
	Yes	180	25.46
	No	527	74.54
Grade level			
	1	212	29.99
	2	164	23.2
	3	186	26.31
	4	74	10.47
	5	71	10.04
Self-rated previous academic performance			
	Poor	118	16.69
	Average	211	29.84
	Good	299	42.29
	Excellent	79	11.17
Voluntarily chose the major			
	Yes	627	88.68
	No	80	11.32
Exposure to AI-medicine interdisciplinary courses			
	Never	43	6.08
	Rarely	128	18.1
	Occasionally	381	53.89
	Frequently	107	15.13
	Very frequently	48	6.79
Received AI training			
	No	516	72.98
	Yes	191	27.02
Interest in AI			
	No	184	26.03
	Yes	523	73.97
Frequency of AI tool use			
	Frequently	371	52.48
	Moderately	272	38.47
	Occasionally	64	9.05
Satisfaction with current major			
	Very dissatisfied	18	2.55
	Dissatisfied	22	3.11
	Neutral	291	41.16
	Satisfied	308	43.56
	Very satisfied	68	9.62
Major			
	Medical Laboratory Technology	21	2.97
	Forensic Medicine	7	0.99
	Stomatology	42	5.94
	Rehabilitation	21	2.97
	Pharmacy	57	8.06
	Basic Medicine	27	3.82
	Biomedical Engineering	21	2.97
	Medical Imaging	31	4.38
	Public Health	13	1.84
	Anesthesiology	8	1.13
	Clinical Medicine	408	57.71
	Nursing	51	7.21

### 3.2 Scoring of AI readiness and AI literacy among medical students

The total AI readiness score of medical students was 78.29 ± 14.09, with a mean item score of 3.56 ± 0.64. The total AI literacy score was 61.08 ± 11.74, with a mean item score of 5.09 ± 0.98. Results are shown in Table 2.

**Table 2:** Scores of medical students’ AI readiness and AI literacy (*n* = 707).

Item	Total score	Average item score
Artificial Intelligence Readiness Scale Cognition Ability Vision Ethics	78.29 ± 14.09	3.56 ± 0.64
	20.34 ± 4.43 36.00 ± 6.64 10.75 ± 2.10 11.21 ± 2.21	3.39 ± 0.74 3.60 ± 0.66 3.58 ± 0.70 3.74 ± 0.74
Artificial Intelligence Literacy Scale Awareness Use	61.08 ± 11.74 15.32 ± 3.07 15.31 ± 3.06	5.09 ± 0.98 5.11 ± 1.02 5.10 ± 1.02
Evaluation	15.10 ± 3.08	5.03 ± 1.03
Ethics	15.35 ± 3.07	5.12 ± 1.02

### 3.3 Latent profile model of AI readiness among medical students

Using the 22 subscale item scores of the Medical Artificial Intelligence Readiness Scale as manifest indicators, models with 1 to 5 profiles were sequentially fitted to determine the optimal model. Model fit results are shown in Table 3. The results indicated that the AIC, BIC, and aBIC values gradually decreased with an increasing number of profiles, with a plateau trend emerging from the three-profile model onward. Among these, the three-profile model exhibited the highest entropy value (0.986), which was higher than that of the four-profile model (0.974) and the five-profile model (0.980), indicating superior classification accuracy. Although the LMR-LRT for the four-profile model was statistically significant (*p* = 0.0468), the four-profile solution predominantly divided the moderate-readiness profile into two subgroups (28.4 and 19.9%) with similar score patterns across the 22 indicators, without yielding a qualitatively distinct or substantively interpretable profile; it also produced a lower entropy and smaller class sizes. The LMR-LRT value for the five-profile model was not statistically significant (*p* = 0.3498), indicating no further improvement over the four-profile model. The average posterior probabilities for all profiles ranged from 0.986 to 0.997, all exceeding 0.85. Combined with classification accuracy results, the three-profile model demonstrated good discriminative ability and high classification precision. Average posterior probabilities are shown in Table 4. Therefore, balancing model fit, classification accuracy, class size, parsimony, and interpretability, the three-profile solution was ultimately selected as the optimal model.

**Table 3:** Model-fit indices for latent profile models of AI readiness among medical students (*n* = 707).

Model	AIC	BIC	aBIC	Entropy	LMRT (*P*)	BLRT (*P*)	Class proportions (%)
1	36642.223	36842.908	36703.198				
2	27795.206	28100.795	27888.055	0.976	0.0000	0.0000	0.556/0.444
3	23868.238	24278.731	23992.960	0.986	0.0001	0.0000	0.484/0.414/0.102
4	22610.587	23125.984	22767.183	0.974	0.0468	0.0000	0.425/0.199/0.284/0.093
5	21368.663	21988.964	21557.132	0.980	0.3498	0.0000	0.044/0.392/0.194/0.280/0.091

AIC, Akaike information criterion; BIC, Bayesian information criterion; aBIC, sample-size-adjusted Bayesian information criterion; LMR-LRT, Lo–Mendell–Rubin adjusted likelihood ratio test; BLRT, bootstrapped likelihood ratio test.

**Table 4:** Average posterior probabilities for the three-profile solution (*n* = 707).

Assigned profile	C1	C2	C3
C1: Low AI readiness	0.993	0.007	0.000
C2: Moderate AI readiness	0.003	0.997	0.000
C3: High AI readiness	0.000	0.014	0.986

C1, low AI readiness; C2, moderate AI readiness; C3, high AI readiness.

### 3.4 Design of latent profiles of AI readiness among medical students

Three latent profiles were identified based on score patterns. C1 (*n* = 342, 48.4%) belonged to the low medical AI readiness profile, with low scores across all items and the lowest overall readiness level. C2 (*n* = 293, 41.4%) was the moderate readiness profile, with item scores in the moderate range. C3 (*n* = 72, 10.2%) was the high readiness profile, with high scores across all items and the optimal overall medical AI readiness level. The three profiles exhibited a stable low-to-high gradient in total medical AI readiness scores and across its four dimensions (Table 5). Figure 1 presents the scores of the 22 items across the three latent profiles.

**Table 5:** AI readiness dimension scores across the three latent profiles (mean ± SD).

Dimension	Total sample (*n* = 707)	C1 (*n* = 342)	C2 (*n* = 293)	C3 (*n* = 72)	*F*	*p*
Cognition	20.34 ± 4.43	17.17 ± 2.29	21.89 ± 2.72	29.03 ± 2.14	791.32	<0.001
Ability	36.00 ± 6.64	30.39 ± 2.68	39.29 ± 2.56	49.21 ± 1.83	2023.54	<0.001
Vision	10.75 ± 2.10	9.15 ± 1.12	11.68 ± 1.22	14.57 ± 1.05	818.32	<0.001
Ethics	11.21 ± 2.21	9.59 ± 1.54	12.23 ± 1.33	14.74 ± 1.71	544.74	<0.001
Total AI Readiness	78.29 ± 14.09	66.30 ± 4.73	85.10 ± 4.79	107.54 ± 4.54	2773.29	<0.001

**Figure 1:**
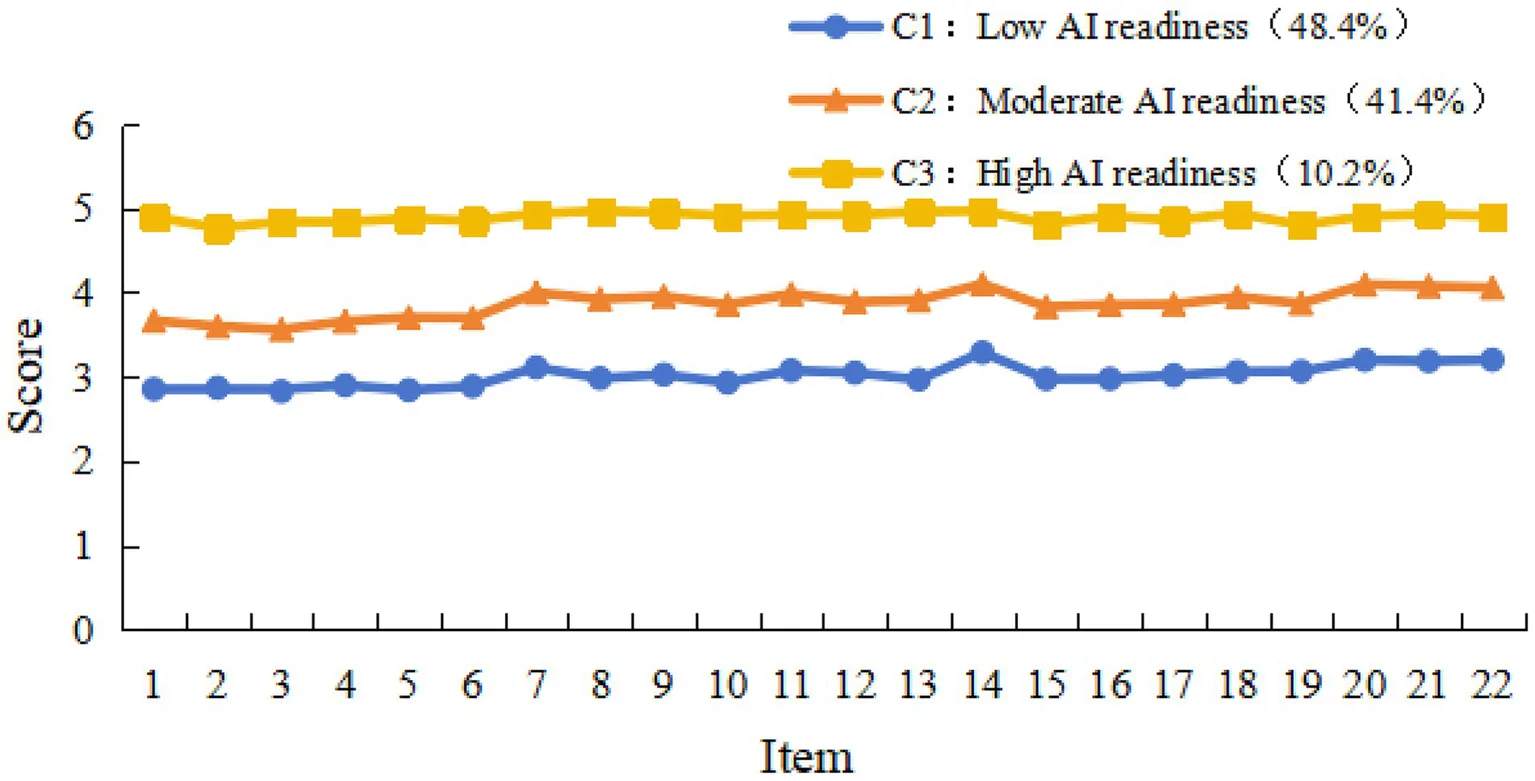
Profile plot of the three latent AI readiness profiles across the 22 manifest indicators. Note: Data points represent the observed item-level mean scores for each indicator within each latent profile. Items are grouped into four C-MAIRS-MS dimensions: Cognition (1–6), ability (7–16), vision (17–19), and ethics (20–22); see Supplementary Table S1 for full item wording.

### 3.5 Univariate analysis of AI readiness characteristics among medical students

Univariate analysis results (Table 6) showed that the three latent classes of medical students differed significantly in gender, place of origin, class cadre status, only child status, self-rated academic performance, voluntary choice of major, exposure to AI-medicine interdisciplinary courses, receipt of AI-related training, interest in AI, frequency of AI tool use, and satisfaction with current major over the past six months (*p* < 0.05). There were no significant differences in grade level or major type. Given the exploratory nature of these univariate comparisons and the relatively large number of tests performed, the associated *p* values should be interpreted descriptively, as an inflated type I error risk cannot be excluded; the subsequent multivariate model served to identify factors independently associated with profile membership.

**Table 6:** Univariate comparisons of participant characteristics across the three latent profiles (*n* = 707).

Variable	Category	Total (*n* = 707)	C1 (*n* = 342)	C2 (*n* = 293)	C3 (*n* = 72)	*χ* ^2^	*p*
Gender	Male	310 (43.84%)	151 (44.15%)	115 (39.25%)	44 (61.11%)	11.244	0.004
	Female	397 (56.16%)	191 (55.85%)	178 (60.75%)	28 (38.89%)		
Place of origin	Rural	390 (55.16%)	203 (59.36%)	153 (52.22%)	34 (47.22%)	10.677	0.030
	Town	197 (27.86%)	95 (27.78%)	83 (28.33%)	19 (26.39%)		
	Urban	120 (16.97%)	44 (12.87%)	57 (19.45%)	19 (26.39%)		
Class cadre	Yes	197 (27.86%)	82 (23.98%)	85 (29.01%)	30 (41.67%)	9.587	0.008
	No	510 (72.14%)	260 (76.02%)	208 (70.99%)	42 (58.33%)		
Only child	Yes	180 (25.46%)	83 (24.27%)	67 (22.87%)	30 (41.67%)	11.259	0.004
	No	527 (74.54%)	259 (75.73%)	226 (77.13%)	42 (58.33%)		
Grade level	1	212 (29.99%)	122 (35.67%)	76 (25.94%)	14 (19.44%)	14.502	0.070
	2	164 (23.20%)	74 (21.64%)	69 (23.55%)	21 (29.17%)		
	3	186 (26.31%)	81 (23.68%)	84 (28.67%)	21 (29.17%)		
	4	74 (10.47%)	38 (11.11%)	29 (9.90%)	7 (9.72%)		
	5	71 (10.04%)	27 (7.89%)	35 (11.95%)	9 (12.50%)		
Self-rated previous academic performance	Poor	118 (16.69%)	71 (20.76%)	33 (11.26%)	14 (19.44%)	25.341	<0.001
	Average	211 (29.84%)	114 (33.33%)	86 (29.35%)	11 (15.28%)		
	Good	299 (42.29%)	129 (37.72%)	136 (46.42%)	34 (47.22%)		
	Excellent	79 (11.17%)	28 (8.19%)	38 (12.97%)	13 (18.06%)		
Voluntarily chose the major	Yes	627 (88.68%)	291 (85.09%)	271 (92.49%)	65 (90.28%)	8.823	0.012
	No	80 (11.32%)	51 (14.91%)	22 (7.51%)	7 (9.72%)		
Exposure to AI-medicine interdisciplinary courses	Never	43 (6.08%)	28 (8.19%)	13 (4.44%)	2 (2.78%)	93.139	<0.001
	Rarely	128 (18.10%)	85 (24.85%)	35 (11.95%)	8 (11.11%)		
	Occasionally	381 (53.89%)	187 (54.68%)	165 (56.31%)	29 (40.28%)		
	Frequently	107 (15.13%)	24 (7.02%)	68 (23.21%)	15 (20.83%)		
	Very frequently	48 (6.79%)	18 (5.26%)	12 (4.10%)	18 (25.00%)		
Received AI training	No	516 (72.98%)	276 (80.70%)	204 (69.62%)	36 (50.00%)	31.299	<0.001
	Yes	191 (27.02%)	66 (19.30%)	89 (30.38%)	36 (50.00%)		
Interest in AI	No	184 (26.03%)	121 (35.38%)	54 (18.43%)	9 (12.50%)	31.167	<0.001
	Yes	523 (73.97%)	221 (64.62%)	239 (81.57%)	63 (87.50%)		
Frequency of AI tool use	Frequently	371 (52.48%)	178 (52.05%)	147 (50.17%)	46 (63.89%)	19.674	0.001
	Moderately	272 (38.47%)	122 (35.67%)	132 (45.05%)	18 (25.00%)		
	Occasionally	64 (9.05%)	42 (12.28%)	14 (4.78%)	8 (11.11%)		
Satisfaction with current major	Very dissatisfied	18 (2.55%)	9 (2.63%)	7 (2.39%)	2 (2.78%)	105.402	<0.001
	Dissatisfied	22 (3.11%)	16 (4.68%)	4 (1.37%)	2 (2.78%)		
	Neutral	291 (41.16%)	186 (54.39%)	93 (31.74%)	12 (16.67%)		
	Satisfied	308 (43.56%)	111 (32.46%)	165 (56.31%)	32 (44.44%)		
	Very satisfied	68 (9.62%)	20 (5.85%)	24 (8.19%)	24 (33.33%)		
Major	Medical Laboratory Technology	21 (2.97%)	10 (2.92%)	9 (3.07%)	2 (2.78%)	30.036	0.118
	Forensic Medicine	7 (0.99%)	2 (0.58%)	4 (1.37%)	1 (1.39%)		
	Stomatology	42 (5.94%)	19 (5.56%)	14 (4.78%)	9 (12.50%)		
	Rehabilitation	21 (2.97%)	13 (3.80%)	5 (1.71%)	3 (4.17%)		
	Pharmacy	57 (8.06%)	27 (7.89%)	22 (7.51%)	8 (11.11%)		
	Basic Medicine	27 (3.82%)	11 (3.22%)	11 (3.75%)	5 (6.94%)		
	Biomedical Engineering	21 (2.97%)	11 (3.22%)	10 (3.41%)	0 (0.00%)		
	Medical Imaging	31 (4.38%)	18 (5.26%)	9 (3.07%)	4 (5.56%)		
	Public Health	13 (1.84%)	10 (2.92%)	3 (1.02%)	0 (0.00%)		
	Anesthesiology	8 (1.13%)	4 (1.17%)	3 (1.02%)	1 (1.39%)		
	Clinical Medicine	408 (57.71%)	193 (56.43%)	185 (63.14%)	30 (41.67%)		
	Nursing	51 (7.21%)	24 (7.02%)	18 (6.14%)	9 (12.50%)		

### 3.6 Multivariate analysis of AI readiness characteristics among medical students

Variables showing significant differences in univariate analysis were entered into a multinomial logistic regression model to identify factors independently associated with profile membership, with variable assignments shown in Table 7. Categorical predictors were entered as sets of dummy-coded indicators with the reference categories specified in Table 7 (i.e., Never for AI-medicine course exposure, No for interest in AI, Frequently for frequency of AI tool use, and Very satisfied for satisfaction with current major), and two logit equations were estimated (C2 vs. C1 and C3 vs. C1). Low AI readiness (C1) was used as the reference outcome category. Regression results revealed that AI-medicine course exposure, interest in AI, frequency of AI tool use, and satisfaction with current major were significantly associated with profile membership (*p* < 0.05). Detailed results are presented in Table 8.

**Table 7:** Variable assignment method.

Variables	Assignment method
Gender	Male = 1, Female = 2
Place of origin	Rural = 1, Town = 2, Urban = 3
Class cadre	Yes = 1, No = 2
Only child	Yes = 1, No = 2
Self-rated previous academic performance	Poor = 1, Average = 2, Good = 3, Excellent = 4
Voluntarily chose the major	Yes = 1, No = 2
Exposure to AI-medicine interdisciplinary courses	Never = 1, Rarely = 2, Occasionally = 3, Frequently = 4, Very frequently = 5
Received AI training	No = 1, Yes = 2
Interest in AI	No = 1, Yes = 2
Frequency of AI tool use	Occasionally = 1, Moderately = 2, Frequently = 3
Satisfaction with current major	Very dissatisfied = 1, Dissatisfied = 2, Neutral = 3, Satisfied = 4, Very satisfied = 5

**Table 8:** Multinomial logistic regression of factors associated with latent profile membership.

Variables	Reference	*β*	SE	Wald *χ*^2^	*p*	OR	95% *CI*
C2 vs. C1							
Exposure to AI-medicine courses	Never						
Very frequently		1.192	0.443	7.234	0.007	3.295	1.382 ~ 7.856
Interest in AI	No						
Yes		0.651	0.210	9.569	0.002	1.918	1.269 ~ 2.897
Frequency of AI tool use	Frequently						
Moderately		0.772	0.359	4.637	0.031	2.164	1.072 ~ 4.370
C3 vs. C1							
Exposure to AI-medicine courses	Never						
Frequently		2.158	0.937	5.308	0.021	8.654	1.380 ~ 54.262
Very frequently		1.936	0.897	4.653	0.031	6.93	1.194 ~ 40.237
Interest in AI	No						
Yes		0.958	0.449	4.547	0.033	2.607	1.081 ~ 6.288
Satisfaction with major	Very satisfied						
Neutral		−2.309	0.482	22.968	<0.001	0.099	0.039 ~ 0.255
Satisfied		−1.084	0.416	6.788	0.009	0.338	0.150 ~ 0.764

Only statistically significant predictors from the final model are displayed.

### 3.7 Association between latent AI readiness profiles and AI literacy of medical students

AI literacy among medical students differed significantly across the three AI readiness levels. Total AI literacy scores and all dimension scores showed a gradient increase from C1 to C3 (all *p* < 0.001), with large effect sizes (*η*^2^ = 0.66 for total AI literacy and 0.56–0.62 across the four dimensions). *Post hoc* pairwise comparisons using the least significant difference (LSD) test revealed that all pairwise differences were statistically significant, with C1 exhibiting the lowest AI literacy and C3 exhibiting the highest AI literacy (Table 9).

**Table 9:** AI Literacy scores across the three latent profiles (mean ± SD).

Profile	Number of cases	Total score	Awareness	Use	Evaluation	Ethics
C1: Low AI readiness	342	52.24 ± 6.64	13.08 ± 1.93	13.06 ± 1.77	12.89 ± 1.89	13.21 ± 1.99
C2: Moderate AI readiness	293	66.51 ± 7.29^a^	16.73 ± 2.00^a^	16.69 ± 2.08^a^	16.40 ± 1.99^a^	16.68 ± 2.13^a^
C3: High AI readiness	72	81.00 ± 6.26^ab^	20.21 ± 1.71^ab^	20.35 ± 1.49^ab^	20.35 ± 1.56^ab^	20.10 ± 1.99^ab^
*F*		675.12	533.95	580.8	572.97	440.6
*p*		<0.001	<0.001	<0.001	<0.001	<0.001

Post hoc pairwise comparisons were performed using the least significant difference (LSD) test. Compared with the low AI readiness group, ^a^*p* < 0.05; Compared with the moderate AI readiness group, ^b^*p* < 0.05.

## 4 Discussion

This study applied latent profile analysis to characterize AI readiness among 707 Chinese medical students and yielded three main findings. First, three distinct AI readiness profiles were identified—low (*n* = 342, 48.4%), moderate (*n* = 293, 41.4%), and high (*n* = 72, 10.2%)—confirming substantial heterogeneity in preparedness. Second, multinomial logistic regression showed that exposure to AI-medicine interdisciplinary courses, interest in AI, frequency of AI tool use, and satisfaction with current major were independently associated with profile membership, whereas demographic variables such as gender and grade level were not. Third, AI literacy increased monotonically across the low-, moderate-, and high-readiness profiles (total scores 52.24 ± 6.64, 66.51 ± 7.29, and 81.00 ± 6.26, respectively; all pairwise *p* < 0.001). The following sections interpret these findings in relation to the previous literature and discuss their implications for medical education.

### 4.1 Heterogeneity of medical AI readiness among medical students

The application of LPA in the present study revealed three distinct subgroups of medical students characterized by low, moderate, and high levels of AI readiness, thereby challenging the assumption of homogeneity within this population. This finding aligns with a growing body of evidence suggesting considerable inter-individual variability in AI preparedness among medical trainees globally (30). Notably, a substantial proportion of students were classified into the low-readiness group, while only a small fraction exhibited high readiness. This distribution is particularly concerning given the rapid integration of AI into clinical practice, which necessitates a workforce adequately prepared to leverage these technologies effectively (7). The observed predominance of students with low readiness resonates with earlier reports indicating that a significant number of medical students lack formal education on AI (31) and exhibit limited readiness for its application in healthcare (32). For example, in a survey of 800 Iranian medical students, the mean total MAIRS-MS score was 70.44 ± 19.32 (13), and Korean medical students using a 7-point version of the same scale scored 4.19 on average, with cognition the lowest subscale (3.92) (14); in the present sample, the corresponding mean total score was 78.29 ± 14.09 (mean item score 3.56 ± 0.64 on a 5-point scale), yet even in this relatively higher-scoring sample, nearly half of the students fell into the low-readiness profile. Many studies using traditional total-score approaches have reported moderate overall readiness levels, which may mask the existence of vulnerable subgroups with significantly lower preparedness (14, 33). The current findings extend this understanding by demonstrating that a large segment of the student population may not be adequately prepared, underscoring the need for targeted educational interventions. Moreover, the identification of a distinct high-readiness group, albeit small, suggests that some students may already possess advanced competencies, pointing to potential peer-learning opportunities. The heterogeneity observed here supports the premise that medical students are not a monolithic group in their readiness for AI, and tailored approaches to curriculum development are necessary to address the diverse needs of learners.

The uniformly low scores observed across all dimensions of medical artificial intelligence readiness within the low-readiness subgroup highlight a critical competency gap extending beyond theoretical understanding, with these students lacking not only a foundational comprehension of AI concepts but also the practical skills required for effective application. This finding aligns with concerns that current educational provisions remain heavily theoretical, offering few opportunities to develop tangible skills in the clinical application of AI (34), and with reports that a majority of medical students receive minimal to no formal education on AI, with hands-on training components rarely included even when such education is offered (5). The absence of robust, hands-on training within medical curricula has been repeatedly identified as a key barrier to developing an AI-capable future workforce, as skills acquisition in this domain is most successful through experiential and applied learning methods (35). The disparities in structured educational opportunities between the low-readiness group and their higher-readiness peers (e.g., 80.7% of the low-readiness profile had never received AI training, compared with 50.0% of the high-readiness profile) further indicate that the ability-dimension deficit reflects systemic curricular gaps rather than individual shortcomings, underscoring the urgent need to embed structured, hands-on AI training in medical education.

The heterogeneous AI readiness profiles identified in this study align with and extend previous research demonstrating variability in how medical students perceive and prepare for AI (30), and with the notion that AI readiness is not a binary attribute but a continuum shaped by a complex interplay of individual and contextual factors. It should be acknowledged that, because the profiles were derived from the same 22 items subsequently used to characterize them, the observed low-to-high gradient is partly inherent in the classification procedure, and the three profiles mainly represent different levels of a shared readiness continuum rather than qualitatively distinct configurations. Even so, the person-centered approach preserves this gradient in a form that directly supports tiered educational planning.

Unlike conventional approaches that predominantly rely on total score comparisons between groups, the present study employed latent profile analysis (LPA) to identify distinct subgroups of medical students with heterogeneous artificial intelligence (AI) readiness characteristics at the individual level. This methodological choice offers a more nuanced understanding of the underlying heterogeneity structure within the population. Traditional variable-centered methods, which assume population homogeneity and focus on aggregate-level associations, may obscure meaningful subgroup differences by averaging across diverse response patterns (36, 37). Indeed, such approaches have been criticized for their limited capacity to capture the complex, multidimensional nature of psychological and behavioral constructs, as they implicitly treat samples as comprising a single, uniform population. In contrast, person-centered analytic techniques, such as LPA, are explicitly designed to uncover latent subpopulations characterized by distinct response profiles, thereby preserving within-group variability and enhancing the precision of inferences.

### 4.2 Factors influencing the latent profiles of medical AI readiness

The multinomial logistic regression analysis revealed that exposure to AI-medicine interdisciplinary courses, interest in AI, frequency of AI tool use, and satisfaction with current major were significantly associated with latent class membership. These findings align with previous research emphasizing the role of structured educational exposure in shaping students’ technological preparedness. Specifically, students who reported more frequent engagement with AI-medicine interdisciplinary courses were significantly more likely to belong to the moderate or high AI readiness profiles compared to the low readiness group. This observation is consistent with earlier work demonstrating that formal curriculum integration is a critical driver of AI readiness among medical trainees (38).

Moreover, the positive association between exposure to AI-medicine interdisciplinary courses and higher readiness levels underscores the value of early, systematic curricular interventions, as medical students often exhibit high enthusiasm but limited formal training in AI applications. The finding that interest in AI independently predicted membership in higher readiness profiles corroborates prior studies indicating that intrinsic motivation and positive attitudes toward AI are closely linked to greater engagement and skill acquisition (39). However, given the cross-sectional design, the direction of these associations remains uncertain: it is equally plausible that students with higher AI readiness are more likely to seek out AI-related courses, engage with AI tools, and report greater academic satisfaction.

Additionally, the significant relationship between greater frequency of AI tool use and higher readiness categories is consistent with the notion that hands-on experience may facilitate the development of practical competencies and confidence in using AI technologies.

Notably, satisfaction with current major was also significantly associated with membership in the high readiness group relative to the low readiness group. This result may reflect a broader engagement with one’s professional training, which could foster a more proactive attitude toward emerging technologies (40). In contrast to some earlier investigations that identified gender or grade-level differences in AI readiness, the present analysis did not observe such demographic variables as significant independent predictors after multivariable adjustment. These discrepancies could be attributed to differences in study populations, measurement instruments, or the analytical approaches employed. The lack of significant association between academic performance and latent class membership further suggests that AI readiness may be more strongly driven by experiential and attitudinal factors than by traditional academic metrics. Collectively, these results reinforce the notion that improving AI readiness among medical students requires multifaceted strategies that integrate structured curricula, foster positive attitudes, and provide ample opportunities for practical application (41).

The relationship between professional satisfaction and AI readiness observed in this study aligns with broader evidence linking affective academic experiences to technology adoption behaviors. Prior research has demonstrated that healthcare professionals’ acceptance of AI-based tools is substantially shaped by their overall professional engagement and work-related attitudes, with those exhibiting greater professional fulfillment showing more favorable orientations toward technological innovation.

### 4.3 The association between AI readiness profiles and AI literacy in medical students

The relationship between professional satisfaction and AI readiness observed in this study aligns with broader evidence linking affective academic experiences to technology adoption, including reports that healthcare professionals with greater professional fulfillment show more favorable orientations toward technological innovation, whereas professional ambivalence or dissatisfaction dampens motivation for engaging with emerging technologies (42). A plausible mechanism is the transfer of learning engagement: students who derive less fulfillment from their academic pursuits may exhibit reduced curiosity and initiative, diminishing their willingness to explore novel tools such as AI. From a practical standpoint, interventions aimed at enhancing professional satisfaction among medical students could therefore yield collateral benefits for AI readiness.

The observation that the latent profiles of AI readiness did not differ significantly across grade levels or major is particularly noteworthy, as it suggests that the progression of medical training itself does not inherently cultivate greater preparedness for AI technologies. This finding aligns with prior research indicating that AI readiness among medical students remains moderate regardless of academic seniority, with some studies even reporting that junior students exhibit higher readiness than their senior counterparts (43, 44), and with the absence of substantial differences in readiness between medical and dental students (44). For example, among 800 Iranian medical students, total MAIRS-MS scores did not differ significantly across school years (68.57 ± 20.47 to 73.22 ± 17.79, *p* = 0.128) (13). Instead, experiential and behavioral factors—such as the frequency of AI tool use, prior exposure to AI-related coursework, and personal interest in the technology—have been consistently identified as stronger correlates of readiness than demographic or educational-stage variables (31, 45), and the lack of a clear educational gradient indicates that structured AI education remains sporadic and often extracurricular (46, 47). This uniformity supports the feasibility of implementing universal, cross-curricular AI literacy programs, while behavior-oriented interventions fostering active engagement with AI are likely to benefit students across all cohorts and disciplines.

## 5 Strengths and limitations

The cross-sectional design of this study precludes any causal inferences regarding the directional relationship between the identified latent profiles of AI readiness and AI literacy among medical students. Our findings demonstrate a clear and consistent gradient, with students in the high AI readiness profile exhibiting the highest AI literacy scores, but the temporal sequence of this association cannot be definitively established. It is plausible that greater AI literacy enhances students’ readiness to engage with AI technologies, potentially by fostering more positive attitudes and reducing anxiety. Conversely, higher AI readiness, characterized by cognitive and ability-related preparedness, may motivate students to acquire more advanced AI literacy, a bidirectional dynamic that has been suggested in previous research. Indeed, prior studies have demonstrated positive correlations between AI literacy and medical AI readiness among health professions students, albeit without determining causality (48, 49). Similarly, research in the nursing field supports an interrelationship among these constructs but is also predominantly cross-sectional in nature (50).

Several methodological considerations warrant attention when interpreting the present findings. Foremost among these is the reliance on self-report measures for both AI readiness and AI literacy, which introduces the potential for social desirability bias. Respondents may have overestimated their competencies to align with perceived expectations regarding AI proficiency, a phenomenon documented in educational technology research where learners tend to inflate their perceived technological capabilities. This tendency is particularly salient in the context of AI, given the increasing emphasis on AI literacy as a core competency for future healthcare professionals (51). Furthermore, the contemporaneous administration of both scales within a single questionnaire battery raises concerns regarding common method bias (CMB), which can artificially inflate the observed associations between constructs. This measurement artifact is a recognized limitation in cross-sectional survey designs that assess multiple psychological attributes simultaneously. Although statistical remedies such as Harman’s single-factor test were considered, they are not a definitive correction for method-induced variance. In addition, the convenience sampling strategy and the restriction of recruitment to four universities in a single province limit the geographic representativeness of the findings and their generalizability to medical students elsewhere in China. The mental-health-related exclusion criterion may have introduced selection bias. Furthermore, assigning participants to their most likely latent profile ignores classification uncertainty, which may result in somewhat underestimated standard errors for the between-profile comparisons, although the very high entropy and posterior probabilities suggest that this effect is minimal.

## 6 Conclusion

The identified heterogeneity in AI readiness among medical students carries significant practical implications for curriculum design and educational policy. Recognizing that students present with distinct preparatory profiles underscores the need for tailored, rather than uniform, educational interventions. This person-centered approach allows educators to allocate resources more effectively, ensuring that foundational training is provided to those with lower readiness while offering advanced, application-oriented opportunities for those already highly prepared. Given that exposure to AI-medicine interdisciplinary courses and received AI training emerged as pivotal factors distinguishing the identified readiness profiles, the development of tiered, precision educational interventions tailored to students’ varying levels of preparedness is strongly warranted. A universal, one-size-fits-all pedagogical strategy is likely to be suboptimal, as it fails to address the heterogeneous learning needs and foundational knowledge gaps that characterize different student subgroups. For students in the low-readiness profile, who may have limited foundational knowledge, targeted foundational courses that demystify AI and build core competencies before progressing to more complex clinical applications could be particularly beneficial. This approach is supported by evidence indicating that a lack of expert training and awareness are common barriers to AI integration in health professions education (52) and that many students lack structured, conceptual knowledge of AI despite expressing interest (53). Therefore, structured introductory modules within the formal curriculum are essential, as students often rely on informal sources like social media for AI information (52). The development of such stratified pedagogical strategies is crucial for cultivating a future medical workforce capable of responsibly integrating artificial intelligence into clinical practice.

## Data Availability

The raw data supporting the conclusions of this article will be made available by the authors, without undue reservation.
