# Supplementary material for: Artificial intelligence readiness and its association with artificial intelligence literacy among Chinese medical students: a latent profile analysis
Source: Front Public Health. 2026 Sep 23;14:1963947. doi: 10.3389/fpubh.2026.1963947 (PMC13642732; doi:10.3389/fpubh.2026.1963947)
Supplement: Supplementary file 1 [file Table_1.docx]

**Supplementary Table S1 Item content of the Chinese version of the Medical Artificial Intelligence Readiness Scale for Medical Students (C-MAIRS-MS).**

| **Item No.** | **Dimension** | **Item content** |
| --- | --- | --- |
| 1 | Cognition | I can define the basic contents of data science. |
| 2 | Cognition | I can define the basic principles of statistics. |
| 3 | Cognition | I can explain how AI systems are trained. |
| 4 | Cognition | I can define the basic concepts and terms of AI. |
| 5 | Cognition | I can properly analyze the data obtained by AI in healthcare. |
| 6 | Cognition | I can differentiate the functions and features of tools and applications related to AI. |
| 7 | Ability | I can organize workflows in accordance with the logic of AI (such as studying, researching, and translation, etc.). |
| 8 | Ability | I can express the importance of data collection, analysis, evaluation and safety for the development of AI in healthcare. |
| 9 | Ability | I can use AI-based information in combination with my professional knowledge. |
| 10 | Ability | I can effectively and efficiently use AI technologies in healthcare services. |
| 11 | Ability | I can use the AI applications according to its purpose. |
| 12 | Ability | I can acquire, evaluate, use, share and create new knowledge through information and communication technologies. |
| 13 | Ability | I can explain how AI applications can provide a solution to a certain problem in the healthcare field. |
| 14 | Ability | I find it very valuable to apply AI to education, services and research purposes. |
| 15 | Ability | I can explain to the patient the AI applications used in healthcare services. |
| 16 | Ability | I can choose the appropriate AI applications for the problems encountered in the healthcare. |
| 17 | Vision | I can explain the limitations of AI technology. |
| 18 | Vision | I can explain the strengths and weaknesses of AI technology. |
| 19 | Vision | I can foresee the opportunities and threats created by AI technology. |
| 20 | Ethics | I can use health data in accordance with laws, regulations and medical ethics norms. |
| 21 | Ethics | I can act in accordance with medical ethical norms while using AI technologies. |
| 22 | Ethics | I can follow regulations regarding the use of AI technologies in healthcare. |
