# Supplementary material for: Artificial intelligence readiness and its association with artificial intelligence literacy among Chinese medical students: a latent profile analysis
Source: Front Public Health. 2026 Sep 23;14:1963947. doi: 10.3389/fpubh.2026.1963947 (PMC13642732; doi:10.3389/fpubh.2026.1963947)
Supplement: Supplementary file 2 [file Table_2.docx]

**Supplementary Table S2 Item content of the Artificial Intelligence Literacy Scale (AIL).**

| **Item No.** | **Dimension** | **Item content** |
| --- | --- | --- |
| 1 | Awareness | I can distinguish between smart devices and non-smart devices. |
| 2 | Awareness | I can identify the AI technology employed in the applications and products I use. |
| 3 | Awareness | I do not know how AI technology can help me. (reverse-scored) |
| 4 | Use | I can skillfully use AI applications or products to help me with my daily work. |
| 5 | Use | I can use AI applications or products to improve my work efficiency. |
| 6 | Use | It is usually hard for me to learn to use a new AI application or product. (reverse-scored) |
| 7 | Evaluation | I can choose the most appropriate AI application or product from a variety for a particular task. |
| 8 | Evaluation | I can evaluate the capabilities and limitations of an AI application or product after using it for a while. |
| 9 | Evaluation | I am familiar with limitations and shortcomings of AI agents. |
| 10 | Ethics | I always comply with ethical principles when using AI applications or products. |
| 11 | Ethics | I am always alert to privacy and information security issues when using AI applications or products. |
| 12 | Ethics | I am always alert to the abuse of AI technology. |
